# In Vitro, Ex Vivo and In Vivo Evaluation of Microcontainers for Oral Delivery of Insulin

**DOI:** 10.3390/pharmaceutics12010048

**Published:** 2020-01-07

**Authors:** Jacob Rune Jørgensen, Feiyang Yu, Ramakrishnan Venkatasubramanian, Line Hagner Nielsen, Hanne Mørck Nielsen, Anja Boisen, Thomas Rades, Anette Müllertz

**Affiliations:** 1 Department of Pharmacy, University of Copenhagen, Universitetsparken 2, 2100 Copenhagen, Denmark; jacob.r.joergensen@sund.ku.dk (J.R.J.); yufeiyang2017@gmail.com (F.Y.); venkatasubramanian.ramakrishnan@sund.ku.dk (R.V.); hanne.morck@sund.ku.dk (H.M.N.); thomas.rades@sund.ku.dk (T.R.); 2 The Danish National Research Foundation and Villum Foundation’s Center for Intelligent Drug Delivery and Sensing Using Microcontainers and Nanomechanics, 2800 Kgs. Lyngby, Denmark; lihan@dtu.dk (L.H.N.); aboi@dtu.dk (A.B.); 3 Department of Health Technology, Technical University of Denmark, Ørsteds Plads, 2800 Kgs. Lyngby, Denmark; 4 Center for Biopharmaceuticals and Biobarriers in Drug Delivery, Department of Pharmacy, University of Copenhagen, Universitetsparken 2, 2100 Copenhagen, Denmark; 5 Bioneer:FARMA, Department of Pharmacy, University of Copenhagen, Universitetsparken 2, 2100 Copenhagen, Denmark

**Keywords:** oral peptide delivery, microdevices, permeation enhancers, protease inhibitors, Caco-2 cells, Franz diffusion cells, oral gavage

## Abstract

Enhancing the oral bioavailability of peptides has received a lot of attention for decades but remains challenging, partly due to low intestinal membrane permeability. Combining a permeation enhancer (PE) with unidirectionally releasing microcontainers (MCs) has previously been shown to increase insulin permeation across Caco-2 cell monolayers. In the present work, this setup was further employed to compare three common PEs—sodium caprate (C_10_), sodium dodecyl sulfate (SDS), and lauroyl carnitine. The concept was also studied using porcine intestinal tissue with the inclusion of 70 kDa fluorescein isothiocyanate-dextran (FD70) as a pathogen marker. Moreover, a combined proteolysis and Caco-2 cell permeation setup was developed to investigate the effect of soybean trypsin inhibitor (STI) in the MCs. Lastly, in vivo performance of the MCs was tested in an oral gavage study in rats by monitoring blood glucose and insulin absorption. SDS proved to be the most potent PE without increasing the ex vivo uptake of FD70, while the implementation of STI further improved insulin permeation in the combined proteolysis Caco-2 cell setup. However, no insulin absorption in rats was observed upon oral gavage of MCs loaded with insulin, PE and STI. Post-mortem microscopic examination of their gastrointestinal tract indicated lack of intestinal retention and optimal orientation by the MCs, possibly precluding the potential advantage of unidirectional release.

## 1. Introduction

Since the discovery of insulin, almost a century ago, there has been a profound pursuit of developing strategies for oral peptide administration in order to improve patient convenience [1,2,3,4]. Many of the approaches reaching clinical trials have been based on combining enteric coatings, protease inhibitors and permeation enhancers (PEs) to ensure gastric and enzymatic protection while improving intestinal absorption [2,4,5]. For overcoming the absorption barrier, PEs have been developed and studied both for paracellular enhancement by tight junction opening and transcellular enhancement for example by perturbation of the enterocyte cell membranes [6]. Recently, the commercial potential using PEs was further cemented with approval by the U.S. Food and Drug Administration of the glucagon-like peptide-1 (GLP-1) agonist, semaglutide, for oral administration based on gastric permeation enhancement by salcaprozate sodium (SNAC) [7]. While commercialization has proved achievable with an oral bioavailability of 0.1–1.2% for peptides such as desmopressin and semaglutide [7,8], there still lies an obvious potential in further improving the fraction of intact peptide absorption. This could lead to lower manufacturing expenses or simply enabling the therapeutic availability of larger macromolecules more prone to degradation in the gastrointestinal (GI) tract. The recent discontinuation of an oral dosage form development of insulin using the PE, sodium caprate (C_10_), confirms this potential as it was deemed commercially unviable due to the high doses required, despite glycemic control similar to subcutaneously administered insulin [9]. The need for high doses is linked to both enzymatic degradation of insulin and diffusion-driven dilution from the site of tablet disintegration, decreasing the co-localization of insulin and C_10_. Evidence from studies of PE-technologies suggests that simultaneous release of PE and peptide is essential for absorption enhancement by creating a local environment of high co-localized concentrations [7,10].

Unidirectionally releasing microdevices have previously been proposed to ensure co-localization of drug and excipients, while simultaneously minimizing the required amount of drug formulation [11,12,13]. Different fabrication technologies have realized a range of such microdevices capable of protecting the drug formulation from the harsh environment in the GI tract until release at the site of absorption [14]. Manufacturing of microcontainers (MCs) has been investigated by several different techniques such as photolithography, hot embossing and mechanical punching resulting in cylindrical reservoirs of 200–300 µm in both height and diameter [15,16,17]. Results from intestinal perfusion studies have indicated spontaneous entanglement of MCs in intestinal mucus, thereby facilitating intestinal retention and thus potentially confining the area of drug absorption [18]. Enhanced local effects of C_10_ together with steep insulin concentration gradients across Caco-2 cell monolayers were recently studied using MCs with fixed optimal unidirectional release towards the cell monolayers [12]. By controlling the distance between MCs and the monolayer, by use of custom-made stands, it was evident that close proximity between the absorptive barrier and the point of release from the MCs is a prerequisite to achieve C_10_-enhanced permeation of insulin [12].

**Figure 1:**
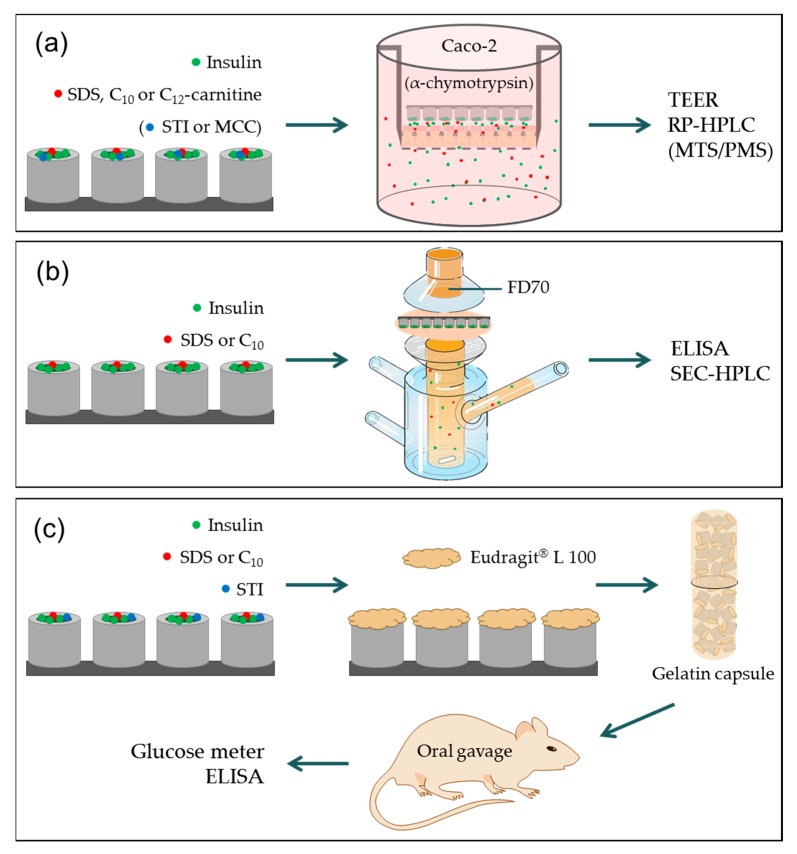
Schematic overview of the studies carried out using microcontainers (MCs). (**a**) In vitro permeation studies of insulin across Caco-2 cell monolayers. SDS: sodium dodecyl sulfate, C_10_: sodium caprate, C_12_-carnitine: lauroyl carnitine, STI: soybean trypsin inhibitor, MCC: microcrystalline cellulose, TEER: transepithelial electrical resistance, RP-HPLC: reversed-phase high-performance liquid chromatography, and MTS/PMS: metabolic viability assay. (**b**) Ex vivo permeation study across porcine intestinal tissue in a Franz diffusion cell setup (illustration edited with permission by PermeGear (Hellertown, PA, USA)). FD70: 70 kDa fluorescein isothiocyanate-dextran, ELISA: enzyme-linked immunosorbent assay, and SEC: size exclusion chromatography. (**c**) In vivo oral gavage studies of coated MCs in a gelatin capsule.

Figure 1 represents a schematic overview of the different studies described in the current work. The initial aim was to extend the previously mentioned distance-controlled in vitro insulin permeation studies across Caco-2 cell monolayers to compare the effect of three common PEs; C_10_, sodium dodecyl sulfate (SDS) and lauroyl (C_12_) carnitine. Further, the potential of loading a protease inhibitor into the MCs was assessed in an in vitro setup combining proteolysis with the barrier properties of Caco-2 cell monolayers. Next, the advantage of unidirectional release was tested in a more physiologically relevant permeation setup by using porcine intestinal tissue and placing PE:insulin loaded MCs on the tissue, in the presence of an apical pathogen marker. Finally, oral in vivo studies were conducted in rats in order to evaluate whether previously published indications on intestinal retention of MCs [18] were sufficient for creating local intestinal environments of high PE and insulin concentrations, leading to the increased oral bioavailability of insulin.

## 2. Materials and Methods

### 2.1. Materials

Single-side-polished Silicon (Si) wafers (N type doping) were acquired from Okmetic (Vantaa, Finland) while the SU-8 constituents (SU-8 2075 and SU-8 Developer) were obtained from Micro Resist Technology (Berlin, Germany). All the following reagents were purchased from Sigma-Aldrich (St. Louis, MO, USA): insulin (human recombinant), sodium dodecyl sulfate (SDS), Dulbecco’s Modified Eagle’s Medium (DMEM), penicillin-streptomycin, l-glutamine (200 mM), non-essential amino acid solution (NEAA, 100×), 4-(2-hydroxyethyl)piperazine-1-ethanesulfonic acid (HEPES), bovine serum albumin (BSA), α-chymotrypsin from bovine pancreas, *N*-benzoyl-l-tyrosine ethyl ester (BTEE), 70 kDa fluorescein isothiocyanate-dextran (FD70) and dibutyl sebacate. *n*-Capric acid sodium salt (C_10_) was procured from abcr (Karlsruhe, Germany) and fetal bovine serum (FBS) from PAA Laboratories (Pasching, Austria). Trifluoroacetic acid (TFA) was purchased from Carl Roth (Karlsruhe, Germany), VIVAPUR^®^ 101 microcrystalline cellulose (MCC) from JRS Pharma (Rosenberg, Germany) and fasted state simulated intestinal fluid (FaSSIF) powder from Biorelevant (London, UK). Eudragit^®^ L 100 was obtained from Evonik (Essen, Germany), lauroyl-dl-carnitine chloride (C_12_-carnitine) was acquired from Chemos (Aldorf, Germany) and hard gelatin capsules, size 9, from Torpac^®^ (Fairfield, NJ, USA). Hanks’ Balanced Salt Solution with calcium and magnesium but without phenol red (HBSS), sodium bicarbonate solution and soybean trypsin inhibitor (STI) powder were bought from Thermo Fisher Scientific (Waltham, MA, USA). Cell viability assay constituents (phenazine methosulfate (PMS) and 3-(4,5-Dimethylthiazol-2-yl)-5-(3-carboxymethoxyphenyl)-2-(4-sulfophenyl)-2H-tetrazolium (MTS)) were purchased from Promega (Madison, WI, USA). Water used throughout the studies was purified by an Ultra Clear UV system (Evoqua Water Technologies, Pittsburgh, PA, USA).

### 2.2. Microcontainer Fabrication, Characterization, Drug Loading and Polymer Coating

SU-8 MCs were fabricated by a two-step photolithography process on Si wafers and subsequently diced into chips (12.8 × 12.8 mm^2^), with each holding 625 MCs, as previously described [15]. MCs used for in vitro and ex vivo studies were developed directly on Si wafers where detachment of individual MCs was not needed. To ensure easy harvest of the MCs for in vivo studies, the MCs were produced on Si wafers with a dedicated release layer made by electron beam deposition of 5 nm of titanium and 20 nm of gold. The dimensions of the MCs on each wafer were determined with an Eclipse L200 bright-field optical microscope (Nikon, Tokyo, Japan) for evaluating the inner and outer diameters, while the inner and overall heights of the MCs were measured by vertical scanning interferometry using a PLu Neox 3D Optical Profiler (Sensofar, Terrassa, Spain). Insulin formulations were loaded as powder mixtures by centrifugal compaction, as described earlier [12]. Furthermore, MCs, loaded for in vivo studies, were sealed with a lid on the opening. This was performed by spray coating a solution of 1% (*w*/*v*) Eudragit^®^ L 100 and 0.54‰ (*v*/*v*) dibutyl sebacate in isopropanol using an ExactaCoat Ultrasonic Spray System (Sono-Tek, Milton, NY, USA) with an infuse rate of 0.05 mL/min, path speed of 5 mm/s and shaping air of 0.02 mbar. In addition, the generator power was set to 2.2 W and an AccuMist nozzle with a spray distance of 50 mm was utilized. Detachment of MCs from the Si-chip for the in vivo studies was carried out with a plastic spatula before loading them into gelatin capsules (size 9) using a ProFunnel (Torpac^®^, Fairfield, NJ, USA). Visualization of the coating and the MCs was carried out by scanning electron microscopy (SEM) both before and after detachment of the Si-chip using a Hitachi TM3030 tabletop microscope (Hitachi High-Technologies Europe, Krefeld, Germany) with an accelerating voltage of 15 kV. The loaded gelatin capsule for oral administration was captured with a Dino-Lite Premier AM7013MZT digital microscope (AnMo Electronics Corporation, Taiwan).

### 2.3. In Vitro Permeation Studies

Insulin permeation was monitored from the apical (1.50 mL) to the basolateral (2.60 mL) compartment across 4.67 cm^2^ polycarbonate Transwell^®^ filters (Corning, Corning, NY, USA) seeded with Caco-2 cells (American Type Culture Collection, Manassas, VA, USA). Cell culturing was done in DMEM supplemented with FBS (10%, *v*/*v*), l-glutamine (2 mM), penicillin (100 U/mL), streptomycin (100 µg/mL) and NEAA (1%, *v*/*v*) for 21–23 days at 37 °C and 5% CO_2_ with a final density of 1 × 10^5^ cells/cm^2^. Three different PEs (SDS, C_10_, or C_12_-carnitine) were loaded into one chip of MCs together with insulin in a 1:4 weight ratio (PE:insulin). The transepithelial electrical resistance (TEER) of the Caco-2 cell monolayers was measured upon equilibration to room temperature using an Epithelial Volt/Ohm Meter (World Precision Instruments, Sarasota, FL, USA) with Endohm™ chambers. First recording took place in culture medium prior to the permeation study; after which, the monolayers were washed twice with permeation buffer consisting of 10 mM HEPES in HBSS (hHBSS) supplied with BSA (0.05%, *w*/*v*), NaHCO_3_ (0.38%, *w*/*v*) and adjusted to pH 7.4. Insulin permeation was monitored for each PE:insulin mixture either loaded into MCs with close contact to the Caco-2 cell monolayer or at a distance of 0.5 mm achieved with custom-made teflon stands as previously described [12]. All three PE:insulin combinations were also tested as solutions of amounts equivalent to the loading capacity of the MC chips (0.6–1.1 mM PE, 0.15 mM insulin) in combination with a chip of empty MCs placed directly on the Caco-2 cell monolayer. Each of the three different studies per PE was carried out in triplicates and across three passages with all studies conducted within eight passages. Insulin permeation was monitored over 2 h at 37 °C and 75 rpm orbital shaking (Compact Shaker KS 15 A, Edmund Bühler, Bodelhausen, Germany) with 100 µL basolateral sampling at 15, 30, 45, 60, 90 and 120 min, and each sampling replaced with 100 µL preheated (to 37 °C) hHBSS. The MC chips were then removed from the Caco-2 cell monolayers, followed by washing twice with hHBSS before TEER was recorded again. Insulin concentrations in the basolateral samples were immediately quantified by reversed-phase high-performance liquid chromatography (RP-HPLC).

### 2.4. Combined In Vitro Permeation and Proteolysis

The effect of various STI concentrations (0, 12, 30, 60 and 120 µg/mL) on α-chymotrypsin inhibition was initially assessed in insulin digestion studies with fixed concentrations of insulin (600 µg/mL) and α-chymotrypsin (10 µg/mL) in FaSSIF buffer at 37 °C. Samples of 240 µL were mixed with 60 µL aqueous TFA solution (5%, *v*/*v*) at 0, 5, 10, 20, 40, 60, 90 and 120 min and analyzed by RP-HPLC to determine the fraction of remaining native insulin. The compatibility of integrating proteolysis in the in vitro permeation setup was then tested by measuring TEER and viability as the metabolic activity of Caco-2 cell monolayers after 2 h of apical exposure to different concentrations of α-chymotrypsin (0, 10, 50 and 100 µg/mL) in hHBSS. Viability was determined after washing the cells with fresh hHBSS followed by the apical addition of 1.5 mL freshly made MTS (240 µg/mL) and PMS solution (2.4 µg/mL) in hHBSS before incubation in dark conditions for 1.5 h with 75 rpm orbital shaking at 37 °C. The absorbance at 492 nm of three 100 µL samples from each well was measured in a 96-well plate using a Multiskan MS 352 Microplate Reader (Labsystems, Helsinki, Finland). Proteolysis was finally incorporated in the in vitro permeation study by the addition of α-chymotrypsin (10 µg/mL) to the apical compartment in an otherwise identical protocol as described in Section 2.3. The only difference was the immediate chymotrypsin inhibition of each basolateral sample by mixing with 25 µL aqueous TFA solution (5%, *v*/*v*). All MC chips were loaded with fixed insulin and SDS amounts of 60% and 20% (*w*/*w*), respectively. The remaining 20% was made up of either STI or MCC.

### 2.5. Ex Vivo Intestinal Permeation Studies

The jejunum and ileum were collected from an anaesthetized pig (approx. 40 kg) immediately before it was euthanized. As the animal was placed under anesthesia and euthanized for a different purpose, no ethical approval was necessary for retrieval of the tissue. Intestinal segments of 10–20 cm were prepared and stored at −20 °C until the day of the permeation study. Prior to the study, a segment was thawed in 10 mM HEPES-buffered DMEM (hDMEM) and longitudinally cut open. Luminal contents were washed away with hDMEM and the muscularis was removed using tweezers before the mucosa was cut into 4 × 4 cm pieces and placed in Franz diffusion cells. Each receptor compartment was filled with 7 mL of hDMEM facing the serosal side of the intestinal tissue. The donor side, facing the mucosa, was filled with 1.5 mL FD70 solution (100 µg/mL) in hDMEM to imitate the inclusion of a pathogen. Permeation was initiated by placing a chip of loaded MCs directly on top with the openings facing the mucosa. Similar to the in vitro permeation study, PE:insulin-loaded MCs were compared to a solution of equivalent amounts of insulin and PE in combination with a chip with empty MCs. Chips with MCs loaded with insulin alone were also included as an additional control group. Insulin and FD70 permeation were monitored over 3 h, with sampling from the receptor compartment at 0, 15, 30, 45, 60, 90, 120 and 180 min with each sampling replaced with an equivalent volume of hDMEM. Quantification of insulin and FD70 was carried out by enzyme-linked immunosorbent assay (ELISA) as described by the supplier (Mercodia, Uppsala, Sweden) and size exclusion high-performance liquid chromatography (SEC-HPLC), respectively.

### 2.6. In Vivo Studies

Male Sprague–Dawley rats (Janvier Labs, Le Genest-Saint-Isle, France) were housed in groups of six per cage and allowed to acclimatize for at least one week with a reversed 12/12 h day/night cycle. Fasting of the rats was initiated 12–14 h prior to the studies, with ad libitum access to water. The rats were 6–7 weeks old and weighed 288 ± 26 g (*n* = 18) on the day of the studies. The experiments were carried out in concordance with the Danish law on animal experiments as approved by the Danish Animal Experiments Inspectorate in accordance with the EU directive 2010/63/EU under license number 2016-15-0201-00892 (25 April 2016). The study was designed with four groups including negative and positive controls receiving subcutaneous (SC) injections of saline or insulin solution (1 IU/kg), respectively, and both oral gavage of empty gelatin capsules. The remaining two groups received SC saline injections and oral gavage of capsules loaded with Eudragit^®^ L 100-coated MCs filled with insulin:PE:STI powder mixtures (6:2:2, *w*/*w*/*w*) using either SDS or C_10_ as the PE. Blood samples of 200 µL were drawn from the tail vein into Microvette^®^ 200 K3E tubes (Sarstedt, Nümbrecht, Germany) at 0, 15, 30, 45, 60, 90, 120, 180 and 240 min. Blood glucose concentrations were determined immediately with a Contour^®^ XT meter (Ascensia Diabetes Care, Basel, Switzerland) before isolating the blood plasma by centrifugation at 9300× *g* for 10 min at 4 °C in a Microcentrifuge 5415 R (Eppendorf, Hamburg, Germany). Plasma samples were then stored at −20 °C until insulin quantification by ELISA (Mercodia, Uppsala, Sweden). Euthanasia of the rats was done in a CO_2_ gassing chamber; after which, the stomachs and small intestines were removed from two of the rats that had been administered MCs in order to localize their position and orientation by fluorescence microscopy, as described in a previous study [19].

### 2.7. HPLC Quantification of Insulin and FD70

A Dionex Ultimate 3000 system (Thermo Fisher Scientific, Waltham, MA, USA) was used for all HPLC analyses of insulin with an injection volume of 20 µL and a column temperature at 22 °C. All insulin samples were quantified as the area under the curve (AUC) of the UV absorbance peak at 214 nm, each time using a new standard curve from 2–100 µg/mL. In vitro permeation samples without proteolysis were separated on a Kinetex XB-C18 column (100 × 4.6 mm, 5 µm, 100 Å; Phenomenex, Torrance, CA, USA), with two mobile phases of A: 0.1% (*v*/*v*) TFA in water and B: 0.1% (*v*/*v*) TFA in acetonitrile. The following gradient was applied with a flow rate of 0.5 mL/min: 0–0.5 min A:B (80:20, *v*/*v*), 0.5–5.5 min A:B (80:20 to 40:60, *v*/*v*), 5.5–6 min A:B (40:60, *v*/*v*), 6–6.5 min A:B (40:60 to 80:20, *v*/*v*) and 6.5–8 min A:B (80:20, *v*/*v*). Samples involving proteolysis were separated on a Luna C18 column (150 × 4.6 mm, 5 µm, 100 Å; Phenomenex, Torrance, CA, USA) with the same two mobile phases using a flow rate of 1.0 mL/min with the following gradient: 0–1.5 min A:B (75:25, *v*/*v*), 1.5–11 min A:B (75:25 to 60:40, *v*/*v*), 11–12 min A:B (60:40 to 20:80, *v*/*v*), 12–13 min A:B (20:80 to 75:25, *v*/*v*) and 13–15 min A:B (75:25, *v*/*v*). FD70 in the ex vivo permeation samples was quantified as the AUC of the fluorescence emission signal of 518 nm with excitation of 492 nm using freshly prepared standards (5–100 ng/mL range) for every analysis. Separation was achieved on a BioSep SEC-s2000 column (300 × 7.8 mm, 5 µm, 145 Å, Phenomenex, Torrance, CA, USA) equipped with a PolySep GFC-P Guard column (35 × 7.8 mm, Phenomenex, Torrance, CA, USA) with a 10 mM phosphate buffer at pH 7.0 as isocratic mobile phase with a flow rate of 1.0 mL/min and a run time of 45 min.

### 2.8. Data Analysis

All data were processed using Microsoft Excel 2010 (Redmond, WA, USA) and GraphPad Prism version 8.2.1 (San Diego, CA, USA) and expressed as the mean ± standard deviation (SD) unless otherwise stated. For cell studies, *n*, determines the number of passages with each passage run in triplicates.

## 3. Results and Discussion

### 3.1. Microcontainer Fabrication and Characterization

MCs fabricated on Si wafers had average outer and inner diameters of 323.2 ± 1.7 and 235.3 ± 1.9 µm, respectively, calculated as the averages of 24 measurements across three wafers by optical microscopy. Overall and inner heights were 250.1 ± 3.1 and 214.0 ± 4.0 µm, respectively, determined with an optical profiler, corresponding to an average loading volume of 9.3 nL per MC.

### 3.2. In Vitro Permeation Studies

**Figure 2:**
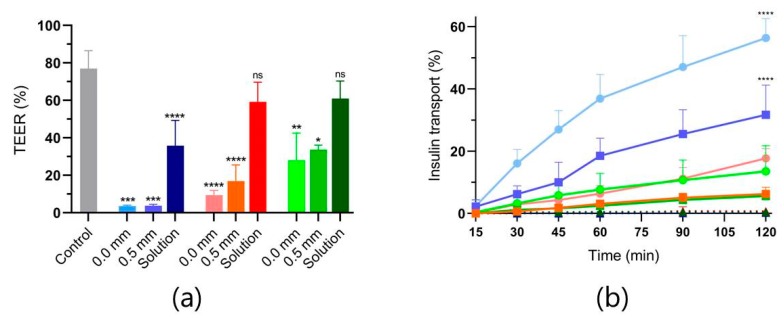
(**a**) Transepithelial electrical resistance (TEER) values of Caco-2 cell monolayers after 2 h permeation enhancer (PE):insulin (1:4, *w*/*w*) exposure at 37 °C relative to their initial value. Blue: SDS, red: C_12_-carnitine, and green: C_10_. Monolayers in the control group were only exposed to fresh permeation buffer. Absolute mean value of initial TEER was 467 ± 34 Ω cm^2^ (*n* = 7). * *p* < 0.05, ** *p* < 0.01, *** *p* < 0.001, **** *p* < 0.0001, and ns: not significant, based on a Tukey’s multiple comparisons one-way ANOVA test comparing TEER after unidirectional release from 0.0 and 0.5 mm with respective solutions and comparing the solutions with the control. (**b**) Accumulated insulin permeation profiles over time. ●: 0.0 mm, ■: 0.5 mm, ▲: solution, blue: SDS, red: C_12_-carnitine, and green: C_10_. **** *p* < 0.0001 based on linear regression analysis by comparing the SDS permeation profiles with the respective permeation profiles of C_10_ and C_12_-carnitine. Data are shown as the mean + SD (*n* = 3).

MC chips were loaded with PE (C_10_, SDS or C_12_-carnitine) and insulin at a 1:4 *w*/*w* ratio. The powder mixtures corresponded to an average loading of 1285 µg insulin and 321 µg PE per MC chip determined by the weight of added powder mixture and insulin quantification by RP-HPLC. Control solutions of each PE:insulin powder mixture, equivalent to the loading capacity of the MC chips, were included in the study in combination with empty MC chips. In this way, all permeation studies were based on equivalent amounts of insulin and PEs. In addition, all Caco-2 cell monolayers were exposed to the presence of MC chips. The results of both TEER and insulin permeation measurements for each study condition are shown in Figure 2.

Confinement of insulin and PE in MCs had significant effects on both TEER values and insulin permeation for all three PEs compared to their respective solutions. As the presence of empty MC chips is not leading to similar insulin permeation, the effect must be due to local high concentrations of insulin and PE. The effect of local high PE concentrations on the monolayer under the opening of C_10_-releasing MCs has previously been visualized by laser-scanning confocal microscopy of cell monolayers cultured on Transwell^®^ filters [12]. Furthermore, we have previously shown that the monolayers, upon local disruptions caused by C_10_-release from MCs, were able to regain almost 90% of initial integrity within 24 h [12]. In the present study, SDS was a significantly more potent PE when compared to C_10_ and C_12_-carnitine, based on the insulin permeation rates. This is in agreement with previous studies where SDS concentrations as low as 0.40 mM resulted in immediate loss of epithelial integrity, while concentrations of C_10_ inducing in vitro permeation usually are in the order of 10 mM [20,21,22]. In comparison, the PE solutions used as controls in the present study were 0.6, 0.7 and 1.1 mM of C_12_-carnitine, SDS and C_10_ respectively. However, the higher potency of SDS also comes with a higher risk of cell toxicity as studies showed that 2 h exposure to 0.40 mM SDS resulted in irreversible deterioration of barrier integrity [21]. The complete loss of monolayer integrity (>95%) when subjected to SDS:insulin-loaded MCs strongly indicates that a substantial number of cells have been washed off the filter. Whether these conditions would result in similar insulin permeation profiles across the more complex intestinal barrier, and if PE-induced pathogen absorption could be a risk, was assessed in the following ex vivo setup.

### 3.3. Ex Vivo Intestinal Permeation Studies

**Figure 3:**
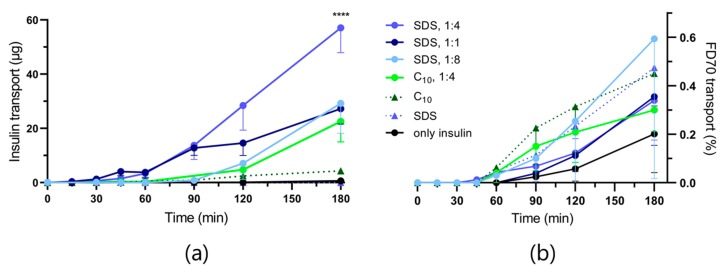
(**a**) Accumulated insulin permeation profiles across porcine intestinal tissue in a Franz diffusion cell setup in buffered Dulbecco’s Modified Eagle’s Medium at 37 °C, ●: loaded MCs with indicated PE:insulin *w*/*w* ratios, ▲: solution of PE and insulin (1:4, *w*/*w*). **** *p* < 0.0001 based on linear regression analysis by comparing the slope from 60–180 min of the permeation profile of MCs loaded with SDS:insulin (1:4, *w*/*w*) with the respective permeation profile of loaded MCs with C_10_:insulin (1:4 *w*/*w*); (**b**) Accumulated FD70 permeation profiles shown as percentages of a total amount of apically applied 150 µg of dissolved FD70. Data are shown as the mean − SD (*n* = 3).

As for the in vitro permeation studies, MC chips were initially loaded with PE (SDS or C_10_) and insulin at a 1:4 *w*/*w* ratio for the ex vivo Franz diffusion cell setup. Comparison was thus only done between C_10_, which had shown equal in vitro potency to C_12_-carnitine, and SDS, which had resulted in significantly higher insulin permeation in vitro. Control solutions corresponding to each PE:insulin powder mixture (1:4, *w*/*w*), (equivalent to the loading capacity of the MC chips), were also included in this study together with an empty MC chip placed directly on the tissue. Furthermore, two additional SDS:insulin ratios (1:1 and 1:8, *w*/*w*) were loaded into MCs to evaluate the potential of optimizing the ratio of SDS and insulin. MCs only loaded with insulin were assessed as a negative control. All insulin permeation profiles across porcine intestinal tissue are shown in Figure 3 together with the corresponding FD70 permeation profiles.

As for the in vitro permeation studies, SDS also resulted in significantly higher insulin permeation across the porcine intestinal tissue compared with C_10_. However, a decrease in insulin permeation was seen for the additional two SDS:insulin ratios (1:1 and 1:8), and no insulin permeation was observed by the MCs without PE. The insulin permeation depends on the interplay between the PE concentration and the insulin concentration gradient experienced by the barrier. An insufficient amount of SDS when loading 1:8 (SDS:insulin, *w*/*w*) could thereby explain the decrease in insulin permeation compared to MCs loaded with 1:4 (SDS:insulin, *w*/*w*). On the other hand, decreasing the insulin concentration gradient across the tissue is likely counteracting the presumably increased effect of SDS, thereby causing lower insulin permeation when loading MCs with 1:1 compared to 1:4 (SDS:insulin, *w*/*w*). Although, none of the tissue barriers were capable of completely retaining FD70 in the donor chamber, no significant differences were observed between any of the FD70 permeation profiles, indicating that the PE exposure did not result in deterioration to a level that allowed for the absorption of pathogens. The Franz diffusion cell setup was chosen as it was optimal for studying unidirectional release on a horizontal barrier. Ussing chambers have previously been used to study lysozyme permeation enhancement by MCs across intestinal tissue [23]. However, the vertical barrier would constitute a challenge regarding controlling the orientation of the MCs. An advantage of the Ussing chamber, however, is the possibility of prolonging tissue viability by using a Krebs-Ringer bicarbonate buffer with a continuous flow of oxygen supply by O_2_:CO_2_ [24]. Storing the tissue samples at −20 °C might have had an impact on their viability and integrity caused by intracellular ice crystal formation [25], and thus FD70 was included as integrity marker. However, previous studies have shown equivalent viabilities between fresh and snap freeze-thawed colorectal tissue samples, yet with poor preservation of integrity [26]. The tissue preparation, including luminal rinsing, might have further reduced the proteolytic activity due to the removal of luminal proteases. Therefore, the development of a simple combined in vitro permeation study with controlled proteolysis was carried out to evaluate the effect of combining permeation enhancing and protease inhibitory excipients in the MCs.

### 3.4. Combined In Vitro Permeation and Proteolysis

**Figure 4:**
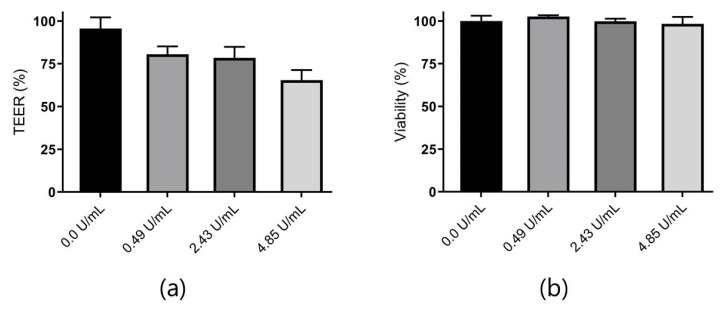
(**a**) Relative TEER of initial values of Caco-2 cell monolayers after a 2 h exposure to various α-chymotrypsin activities in permeation buffer at 37 °C. The absolute mean value of initial TEER was 345 Ω cm^2^, calculated based on twelve wells from one passage (*n* = 1); (**b**) The viability of Caco-2 cell monolayers relative to control cells (0 µg/mL) determined by an MTS/PMS assay immediately after α-chymotrypsin exposure for 2 h. Data are shown as the mean + SD, calculated based on three wells from one passage (*n* = 1).

α-Chymotrypsin activity was initially determined as 48.5 U/mg, following the procedure for enzymatic assay using BTEE as a substrate as described by the supplier [27]. Caco-2 cell monolayers were then exposed to different concentrations of α-chymotrypsin for the assessment of compatibility of combining permeation and proteolysis studies. TEER measurements and the relative viability of the monolayers after a 2 h exposure are shown in Figure 4.

**Figure 5:**
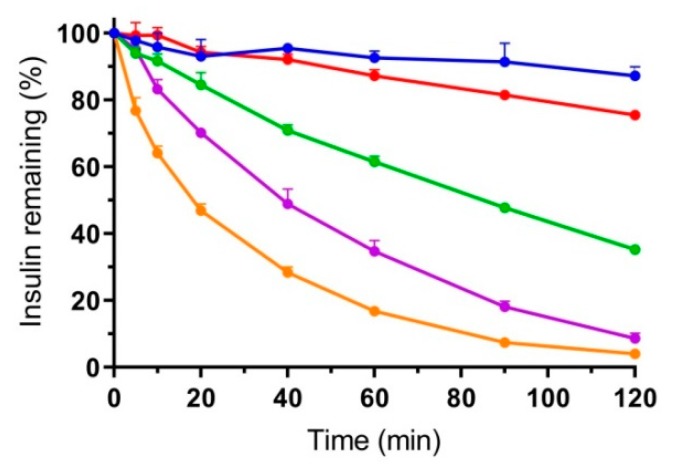
Digestion profiles of insulin (600 µg/mL) by α-chymotrypsin (0.49 U/mL) in fasted state simulated intestinal fluid (FaSSIF) at 37 °C with different concentrations of STI. Orange: 0 µg/mL, purple: 12 µg/mL, green: 30 µg/mL, red: 60 µg/mL and blue: 120 µg/mL. Data are shown as the mean + SD (*n* = 3).

None of the tested α-chymotrypsin concentrations influenced the viability of the Caco-2 cells. However, TEER was decreased by 15% when exposed to 0.49 U/mL compared to the control group without α-chymotrypsin, indicating a relatively small effect on the tight junctions in the cell monolayer. Previous studies have described similar findings when subjecting Caco-2 cell monolayers to different proteolytic enzymes and further documented the reversibility of this effect [28]. For these reasons, studies to determine the effect of STI on inhibiting insulin digestion by α-chymotrypsin were initiated. Based on the previously published average loading capacity of 1.2 mg per MC chip [12] (equal to 960 µg of insulin with a 1:4 (*w*/*w*) PE:insulin loading); insulin concentrations were fixed at 600 µg/mL for all initial digestion studies, corresponding to complete release from the MCs in an apical volume of 1.50 mL. The concentration of α-chymotrypsin was set at 10 µg/mL (0.49 U/mL), based on the suggested ratio of α-chymotrypsin:peptide (1:60, *w*/*w*) for peptide digestion studies by the supplier, as well as previously published work on insulin digestion using a similar amount of α-chymotrypsin (8 µg/mL) [29,30]. Insulin digestion profiles with the addition of different STI concentrations are shown in Figure 5.

**Figure 6:**
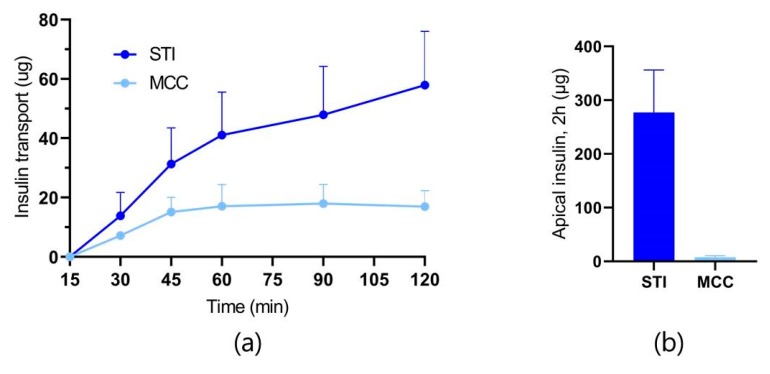
(**a**) Insulin permeation profiles across Caco-2 cell monolayers with MCs loaded with insulin:SDS:STI or insulin:SDS:MCC (both 6:2:2, *w*/*w*/*w*) and placed at a distance of 0.5 mm from the monolayer in permeation buffer with apical α-chymotrypsin (0.49 U/mL) at 37 °C; (**b**) Amounts of native insulin left on the apical side after 2 h of combined permeation and proteolysis study. Data are shown as the mean + SD, calculated based on three wells from one passage (*n* = 1).

Inhibition of α-chymotrypsin was observed even with the addition of only 12 µg/mL of STI, equivalent to 2% (*w*/*w*) relative to insulin, yet more than 90% of native insulin was still degraded after 2 h. With the addition of 120 µg/mL of STI, corresponding to 20% (*w*/*w*) relative to insulin, only 13% digestion was observed over 2 h. Therefore, a combined permeation and proteolysis study was initiated with MCs loaded with equivalent amounts of SDS (20%, *w*/*w*) as in the in vitro permeation studies, and 20% (*w*/*w*) of either STI or MCC, leaving 60% (*w*/*w*) filling capacity of the containers left for insulin loading. The distance between MCs and the monolayer was fixed at 0.5 mm to allow for better insulin accessibility by α-chymotrypsin, resulting in the permeation profiles shown in Figure 6.

Despite the simplicity of the in vitro model, only accounting for one type of cells and one type of proteases, the absorption-enhancing effect as a result of protease inhibition by STI is clearly observed. Only approximately 20% native insulin is remaining after 45–60 min in the initial digestion study (Figure 5), which is at the same time interval that insulin permeation ceases for the group without STI (Figure 6). Concurrently, the insulin gradient is maintained for the group including STI resulting in a 3.4-fold higher insulin transport after 2 h than without STI. Almost all the insulin was digested on the apical side after 2 h in the absence of protease inhibition, while the presence of STI evidently allowed for further insulin uptake (Figure 6). Since the results obtained from the in vitro and ex vivo studies were all obtained with fixed optimal unidirectional release from the MCs, an in vivo study was necessary to evaluate whether the intestinal retention and orientation of MCs in the GI tract were sufficient to achieve absorption of insulin upon oral administration.

### 3.5. In Vivo Studies

**Figure 7:**
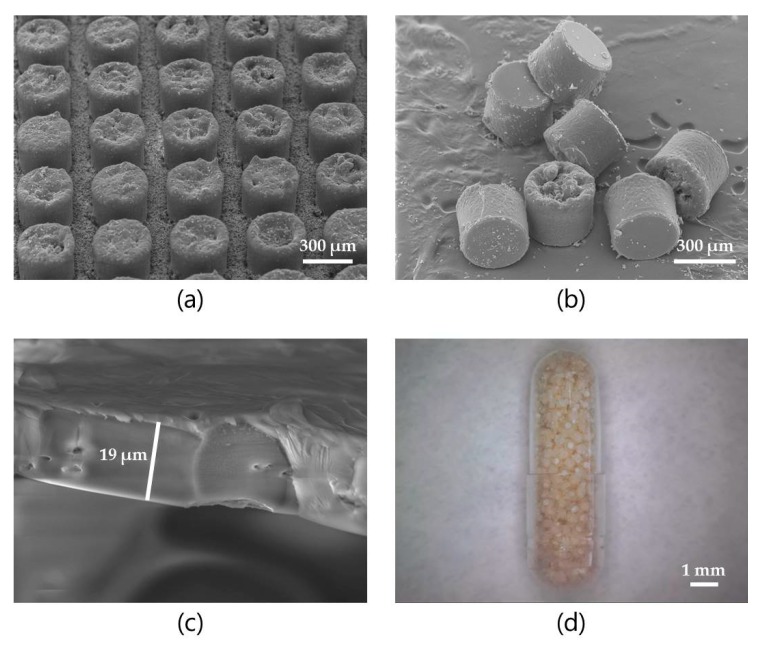
(**a**) Scanning electron microscopy (SEM) image of part of a Si-chip with Eudragit^®^ L 100 coated MCs; (**b**) SEM image of detached MCs; (**c**) SEM image of the coating (thickness approximately 19 µm); (**d**) Micrograph of a size 9 gelatin capsule filled with coated MCs.

After loading of MCs with insulin:SDS:STI or insulin:C_10_:STI (both 6:2:2, *w*/*w*/*w*) and coating the openings of the MCs with Eudragit^®^ L 100, visualization of the coating thickness and the MCs was carried out as shown in Figure 7a–c. The final dosage form for oral gavage, a size 9 gelatin capsule filled with coated MCs, is shown in Figure 7d.

**Figure 8:**
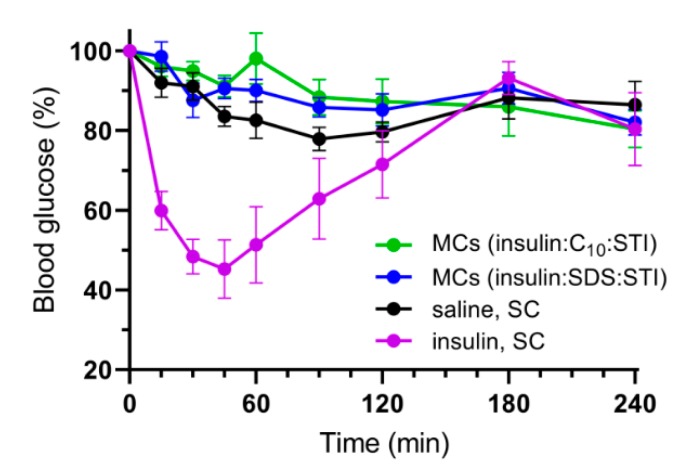
Change in blood glucose in rats over 4 h after oral gavage of MCs loaded with insulin (104 IU/kg), STI and either C_10_ or SDS compared to subcutaneous (SC) injections of either saline or insulin in saline (1 IU/kg). Data are shown as the mean ± standard error of the mean (*n* = 4–5).

The total insulin doses in the size 9 capsules were determined from the loaded mass per capsule and by weighing MC chips before and after detachment giving an average weight of 18.82 µg per coated MC. Based on these calculations; the rats were orally administered 673 ± 20 MCs corresponding to 104 ± 8 IU/kg of insulin and 1.20 ± 0.09 µg/kg of both PE and STI. Figure 8 shows the change in blood glucose measured over 4 h for the two groups receiving MCs by oral gavage and for the negative and positive controls receiving SC injections of saline and insulin in saline (1 IU/kg), respectively.

Neither the change in blood glucose nor detection of insulin by ELISA showed any signs of insulin absorption from the MCs. Previous studies in Caco-2 cell monolayers clearly showed that close proximity between MCs and the epithelial barrier is a necessity in order to enable permeation enhancement of insulin even with fixed optimal unidirectional release [12]. While the ex vivo intestinal permeation studies showed the potential of unidirectional release for permeation enhancement across intestinal tissue, this effect was not observed in vivo. Fluorescent microscopic examination of the stomachs and small intestines of the rats after the in vivo study revealed that all MCs had undergone complete transit from ileum to caecum after 4 h, which is consistent with another recent study of biodegradable MCs [31]. Previous studies have shown SU-8 MCs embedded in the intestinal mucus in in situ intestinal perfusion studies [18]. However, the present study could not confirm this behavior. Instead the in vivo data indicate that the MCs do not achieve sufficient intestinal retention and orientation necessary for realizing insulin absorption. The need for optimizing the in vivo behavior of MCs is further substantiated when considering the aspect of drug loading capacity, as the loaded drug formulation accounts for only 14% of the total weight of a coated MC. Thereby, the effect gained from the unidirectional release must increase bioavailability by more than 7-fold in order to advantageously compensate for the additional drug formulation, which could be fitted in a capsule by excluding the spatial limitation of the MC-material. Current work is therefore looking into improving both intestinal orientation and retention by structural designs, surface engineering and functional polymer coatings of the MCs, which might eventually make it possible to increase the oral bioavailability of insulin and other macromolecules [19,23,32].

## 4. Conclusions

In the present study, we have investigated the concept of enhancing insulin permeation by co-localizing insulin and PE through unidirectional release from MCs. SDS proved more efficient as PE than C_10_ and C_12_-carnitine, increasing insulin permeation across both, Caco-2 cell monolayers and porcine intestinal tissue. The increased insulin permeation achieved ex vivo did not compromise the barrier properties towards the apically included pathogen marker, FD70. A combined proteolysis and permeation study suggested an advantage of including STI in the MCs. However, the MCs did not increase in vivo insulin absorption upon oral gavage to rats, due to a lack of sufficient retention and orientation of MCs in the intestinal mucus. Further strategies in order to achieve close proximity between MCs and enterocytes with optimal unidirectional release in vivo will thus be the focus of future studies.
